# Optimizing sgRNA length to improve target specificity and efficiency for the GGTA1 gene using the CRISPR/Cas9 gene editing system

**DOI:** 10.1371/journal.pone.0226107

**Published:** 2019-12-10

**Authors:** Anders W. Matson, Nora Hosny, Zachary A. Swanson, Bernhard J. Hering, Christopher Burlak

**Affiliations:** 1 Schulze Diabetes Institute, Department of Surgery, University of Minnesota School of Medicine, Minneapolis, MN, United States of America; 2 Department of Medical Biochemistry and Molecular Biology, Suez Canal University, Faculty of Medicine, Ismailia, Egypt; University of Florida, UNITED STATES

## Abstract

The CRISPR/Cas9 gene editing system has enhanced the development of genetically engineered animals for use in xenotransplantation. Potential limitations to the CRISPR/Cas9 system impacting the development of genetically engineered cells and animals include the creation of off-target mutations. We sought to develop a method to reduce the likelihood of off-target mutation while maintaining a high efficiency rate of desired genetic mutations for the GGTA1 gene. Extension of sgRNA length, responsible for recognition of the target DNA sequence for Cas9 cleavage, resulted in improved specificity for the GGTA1 gene and less off-target DNA cleavage. Three PAM sites were selected within exon 1 of the porcine GGTA1 gene and ten sgRNA of variable lengths were designed across these three sites. The sgRNA was tested against synthetic double stranded DNA templates replicating both the native GGTA1 DNA template and the two most likely off-target binding sites in the porcine genome. Cleavage ability for native and off-target DNA was determined by *in vitro* cleavage assays. Resulting cleavage products were analyzed to determine the cleavage efficiency of the Cas9/sgRNA complex. Extension of sgRNA length did not have a statistical impact on the specificity of the Cas9/sgRNA complex for PAM1 and PAM2 sites. At the PAM3 site, however, an observed increase in specificity for native versus off-target templates was seen with increased sgRNA length. In addition, distance between PAM site and the start codon had a significant impact on cleavage efficiency and target specificity, regardless of sgRNA length. Although the in vitro assays showed off-target cleavage, Sanger sequencing revealed that no off-target mutations were found in GGTA1 knockout cell lines or piglet. These results demonstrate an optimized method for improvement of the CRIPSR/Cas9 gene editing system by reducing the likelihood of damaging off-target mutations in GGTA1 knocked out cells destined for xenotransplant donor production.

## Introduction

Xenotransplantation could provide a much-needed source of donor organs for use in the treatment of a wide array of human diseases. The success of xenotransplantation has historically been limited by high rates of xenograft rejection, including hyperacute rejection, acute humoral xenograft rejection, immune cell-mediated rejection, and chronic rejection[1]. Xenotransplant recipients must remain on immunosuppressant drugs indefinitely in order to prevent rejection and extend the lifespan of the xenotransplant. Pigs are considered appropriate candidates for xenotransplantation to humans due to similarities in organ size and metabolism, however, multiple human genetic mutations produce phenotypes known to cause immune responses leading to xenograft rejection[1]. The most commonly targeted porcine genes include α-1,3-galactosyltransferase (GGTA1), cytidine monophosphate-N-acetylneuraminic acid hydroxylase (CMAH), and β-1,4N-acetylgalactosaminyltransferase (β4GalNT2) [1]. Recent advances in precision gene editing have allowed for the successful development of genetically engineered (GE) porcine donors targeting these genes for greater compatibility with the human immune system in order to reduce the likelihood of xenograft rejection[1].

### GGTA1 knockout

Galactose-α-1,3-galactose (α-Gal) epitopes on the surface of porcine cells are produced by the α-1,3-galactosyltransferase enzyme (*GGTA1*). Disruption of the GGTA1 gene, resulting in the production of an α-Gal KO (GTKO) pig, has been shown to improve xenograft survival[2]. In 2014, using the clustered regularly interspersed palindromic repeats (CRISPR) and CRISPR associated protein 9 (Cas9) gene editing system, Burlak et al. found that human immunoglobulin M (IgM) and immunoglobulin G (IgG) antibodies demonstrated reduced binding to peripheral blood mononuclear cells (PBMCs) obtained from both GGTA1 KO and GGTA1/CMAH double-KO pigs[3]. Estrada et al. found that human and primate IgM and IgG antibody binding to PBMCs was reduced in triple KO pigs for GGTA1, CMAH, and β4GalNT2[4]. Thus, GTKO is a necessary step for the production of porcine donors for xenotransplantation.

### Gene editing technologies and limitations

In the last decade, a superior method of gene-editing allowing for precise genetic modification has been developed: the CRISPR/Cas9 gene editing system. First identified by Doudna et al. in 2012[5], the CRISPR/Cas9 system takes advantage of an existing defense mechanism employed by bacteria upon viral attack[5–7]. Prior to the emergence of CRISPR/Cas9[5], gene editing was accomplished by less efficient, programmable nucleases: zinc finger nucleases (ZFNs) and transcription activator-like effector nucleases (TALENs)[8]. Although CRISPR/Cas9 is more efficient compared to these methods[8], a potential limitation common among all gene editing systems is the formation of unwanted off-target mutations (OTM)[9]. OTM occurring with the use of CRISPR/Cas9 have been well documented[10]; although OTM are not problematic for bacteria, they may impact the successful development of GE porcine donors for xenotransplantation[11]. OTM occur when the single guide RNA (sgRNA)/Cas9 complex binds to mismatched DNA sequences in the genome and create double stranded DNA breaks within these regions[9]. According to Fu et al., the use of 20 base pair (bp) sgRNA results in frequent unintended mutations occurring in complementary sequences with up to five mismatches[10]. Occurrence of OTM in GE cells, intended for use in functional assays or in the development of piglets for xenotransplantation, could lead to cells with altered metabolisms and phenotypes, giving rise to skewed results. Thus, it is especially important that OTM are taken into consideration when designing sgRNA for use in GE. We hypothesized that use of sgRNA targeting the GGTA1 gene, designed to exceed the conventional 20 bp spacer region length, would result in increased specificity and decreased OTM while maintaining high cleavage efficiency for the targeted GGTA1 DNA sequence. Reduction of OTM would decrease the likelihood of associated negative impacts on the metabolism and phenotype of GE cells for xenotransplantation.

## Materials and methods

### sgRNA design

**Fig 1:**
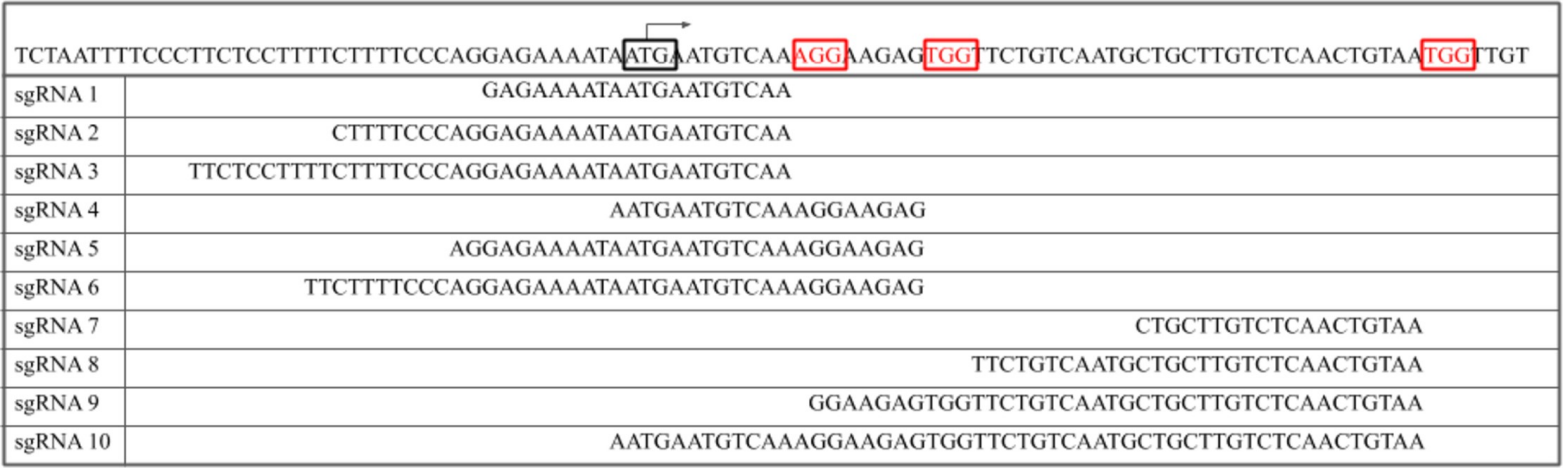
Design of optimized sgRNA. Sequences for each of the 10 sgRNA created and used in this study are shown; sgRNA are aligned against the exon 1 region of the *GGTA1* DNA sequence. The start codon (Methionine) is boxed with a transcription start arrow indicating its position. Red text and red boxes indicate the three PAM sites. At each PAM site, sgRNA DNA sequence and alignment with the GGTA1 DNA sequence are shown.

In designing sgRNA for this experiment, exon 1 of the GGTA1 gene was targeted for CRISPR/Cas9 cleavage. Possible target sites within the GGTA1 gene were identified to design optimized and efficient sgRNA. The GGTA1 sequence was screened for selection of potential target cut sites using the ZiFiT Targeter software(http://zifit.partners.org/ZiFiT)[12,13] and three protospacer adjacent motif (PAM) sites were selected and designated as PAM1, PAM2 and PAM3. In total, 10 sgRNA were designed for testing across the 3 PAM sites; sgRNA for PAM1 and PAM2 were designed with 20, 30, and 40 bp spacer lengths, while sgRNA designed for PAM3 was designed with 19, 30, 40 and 53 bp spacer lengths (Fig 1). Following guide selection, the Guide-it sgRNA *In Vitro* Transcription Kit and Guide-it sgRNA Screening Kit (Takara Bio Inc.)[14] was used to create optimized sgRNA for evaluation of target cleavage efficiency according to the protocol provided by the manufacturer[14].

### Validation of RNP formation and *in vitro* cleavage of genomic GGTA1 DNA template

All sgRNAs were synthesized, isolated, and purified in preparation for testing against the GGTA1 DNA template. To confirm successful formation of Cas9/sgRNA ribonucleoprotein (RNP) complex and subsequent cleavage of genomic GGTA1 DNA template, an 1800 bp sequence of genomic porcine DNA was PCR amplified for the GGTA1 gene and PCR primers were designed such that the cut site (PAM) was located asymmetrically within the template. Asymmetric cleavage allowed for identification of the two cleavage fragments and any remaining uncleaved fragment. In our study, sgRNA spacer length was intentionally varied, therefore, a molecular ratio was calculated for each guide to ensure that an equal amount of guide was available for each reaction. The RNA concentration of each guide was also determined in preparation for cleavage assays to confirm that an equal molecular amount of RNA was used for each assay. Absorbance ratios of 260/280 and 260/230 were calculated prior to use to confirm that RNA was not degraded and of the proper quality. For each of the guide lengths evaluated in this experiment, the actual amount of RNA used is shown (Table 1). The sgRNA was combined with the Cas9 enzyme to create an activated RNP complex to be combined with the GGTA1 DNA template for subsequent cleavage assay according to the *in vitro* transcription protocol provided by the manufacturer (Takara Bio Inc)[14]. A C1000 Touch^TM^ Thermal Cycler (Biorad) was used for all steps. Following completion of cleavage assay, the resulting cleavage assay products were run on a 2% agarose gel to determine cleavage potential at the DNA target site. Control DNA (614bp template) and highly specific sgRNA towards a non-GGTA1gene were used in the experiment to determine if the Cas9 enzyme was functional. The sgRNA used for controls were optimized for >95% cleavage of the control DNA fragment. Negative controls included only DNA template, no Cas9 enzyme was added. The same control reagents were used for all in vitro cleavage assays. The following primers were used in this study to amplify the 1600bp sequence of the GGTA1 gene from genomic porcine DNA.

Forward 5’ GAAGTGGCAGAGCCTAGATTAT 3’

Reverse 5’ AAGAAAGTCCAAGCGGTATCA 3’

**Table 1:** Mass of sgRNA used to conduct *in vitro* cleavage assays.

sgRNA Length (bp)	Mass RNA (ng)
19	47.5
20	50
30	75
40	100
53	132.5

Mass of RNA used for each reaction depended on the length of the sgRNA; 0.4ng of RNA was used per bp of sgRNA.

### Off-target DNA binding site template design and creation

ZiFit Targeter software[12,13], and CAS-OFFinder software (http://www.rgenome.net/cas-offinder/)[15] were used to identify possible off-target binding sequences for the three PAM sites in order to determine the likelihood of off-target cleavage (OTC). The list of possible off-target sequences were screened for matches in the porcine genome by running a basic local alignment search tool (BLAST) analysis (NCBI; https://blast.ncbi.nlm.nih.gov/Blast.cgi). Off-target sites that most closely matched genes found in the porcine genome were selected. For each of the PAM sites, two off-target sequences were selected and used to design 600 bp synthetic double-stranded DNA (dsDNA) fragments (gBlocks® Gene Fragments, IDT) with 1–3 bp mutations (Table 2). Non-mutated native GGTA1 DNA template was created by the same method for comparison of OTC and cleavage targeting the native, non-mutated GGTA1 sequence.

**Table 2:** DNA template design for off-target binding sites.

PAM Site	Template ID	DNA Sequence
PAM1	Native	GAGAAAATAATGAATGTCAA
PAM1	Off-Target #1	GAGAAAATAAT**T**AATGT**A**A**G**
PAM1	Off-Target #2	GAGAAAATAATGAATGT**TC**A
PAM2	Native	AATGAATGTCAAAGGAAGAG
PAM2	Off-Target #1	AATGAATGTCAA**T**GGAAG**T**G
PAM2	Off-Target #2	AATCAATGTCAAA**T**GAAGAG
PAM3	Native	GCTGCTTGTCTCAACTGTAA
PAM3	Off-Target #1	GCTGCTTGTCTCAACT**A**TAA
PAM3	Off-Target #2	GCTGCTTG**G**CTC**T**ACTGTAA

The native sequence remained constant across all PAM sites and the unmutated region of each PAM site is shown for reference. Mutated bases within the 20 bp spacer region preceding the PAM site are shown bolded and underlined.

### Testing the specificity of sgRNA for the native versus off-target DNA templates

All 10 sgRNAs were tested against each of the dsDNA templates created for their corresponding PAM site using the in vitro cleavage assay referenced above [14]. Each experiment was performed in 3 separate reactions to ensure reproducibility. The RNPs were made new each time an assay was performed. The cleavage products that resulted after each assay were then run on a 2% agarose gel to determine the RNPs ability to cleave the various synthetic DNA templates. The data was then analyzed and averaged.

### Densitometric analysis of *in vitro* cleavage assay products

To determine the percent cleavage of each DNA template by its corresponding sgRNA, all gels that were produced for each PAM site were analyzed using densitometry. To establish the percentage of the DNA templates that were cleaved by the sgRNA/Cas9 RNPs, a total signal for the three DNA bands was calculated for each guide by gating at ~ 600 bp, ~400 bp, and ~200 bp. Gates were kept the same size across each gel in order to avoid error and preserve the same signal area for each reading. The total percent cleaved was calculated by dividing the 400 and 200 bp signals by the total signal from all three bands according to the following equation:

$$\frac{Cleaved\;Fragments\;Signal}{Total\;Signal}=Template\;Cleavage\;(\%)$$

The mean percent cleaved and uncleaved was then generated by averaging the values for all replicates for each PAM site, each reaction was run a minimum of three times. This averaged data was used to determine the cleavage specificity and efficiency for all sgRNAs.

### Sanger sequencing of potential off-target sites

Genomic DNA was isolated from the WT porcine fetal fibroblasts (PFF), GTKO transfected PFF, GTKO piglet tail fibroblasts (produced and procured under the University of Minnesota IACUC 1711-35291A) using the QIAamp DNA Blood Mini Kit (QIAGEN, Cat. No. 51304), according to manufacturer’s instructions. DNA concentration and purity at the absorbance ratio 260/280 and 260/230 were determined on a NanoDrop 2000c Spectrophotometer (Thermo-Fisher Scientific). Using ZiFit targeter software[12,13] and Cas-OFFinder [15] to detect potential off-target sites, eight sets of primers were designed to amplify the 8-potential off-target sites (S1 Table) from the genomic DNA using the online Primer Quest tool (Integrated DNA Technologies) based on available sequences obtained from the NCBI GenBank database and double checked by in-silico PCR amplification using NCBI primer blast tool. Primers and products sizes are shown in (S2 Table). Off-target sites were chosen with and without adjacent PAM sites in order to include more possible off-target binding sites. All PCR reactions were initiated at 95°C for 30 seconds, followed by 35 cycles of 95°C for 15 seconds, 56–62°C for 30 seconds, and 68°C for 60 seconds. Reactions were terminated for 10 minutes at 68°C. PCR products were purified by PCR Cleanup kit (Macherey-Nagel GmbH & Co, Ref. 740609.250).

To detect indels in the amplified off-target sites, WT PFF, transfected PFF, GE pig cell PCR products were Sanger sequenced then aligned using SnapGene (Version 5.0.4). The aligned sequences that match upstream and downstream of the target site evaluated for mutations.

### Statistical analysis

R Studio was used for all statistical analysis conducted in this experiment. Differences in percent template cleavage were determined by Two-Way ANOVA Factorial Analysis. Standard error of the mean is reported. Significant value is defined as (p<0.05).

## Results

### Longer sgRNA are able to form functional RNPs and cleave DNA targets

**Fig 2:**
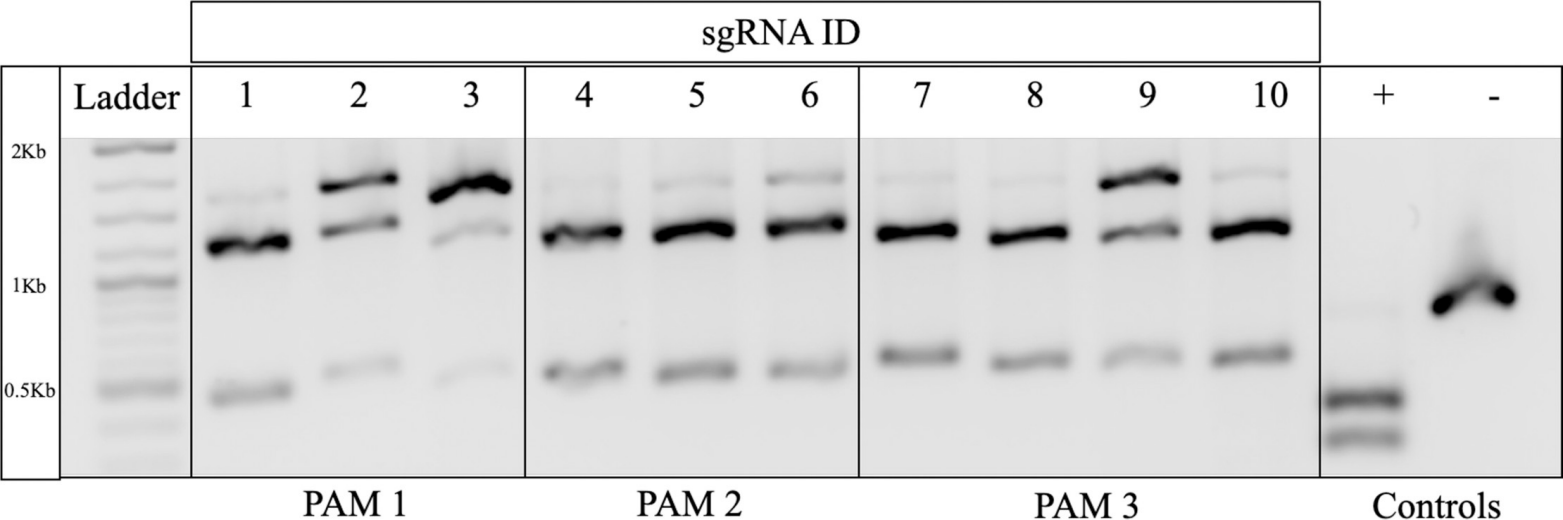
Results of sgRNA/Cas9 mediated cleavage of genomic DNA template. To confirm that longer sgRNAs were capable of forming functional RNPs that are capable of creating double stranded DNA breaks in to their target gene, an in vitro cleavage assay was performed. sgRNA was combined with Cas9 enzyme and added to genomic porcine DNA that was PCR amplified around a 1600 bp region of the GGTA1 gene. The PAM site was located asymmetrically within the DNA fragment to allow for visualization of two cleaved fragments of DNA ~1200bp, ~400bp. All 10 sgRNA designed for the targeting of GGTA1 were able to form a functional RNP that targeted the GGTA1 gene. Uncleaved DNA template is shown as the largest band appearing on top of the two other fragments. The two smaller bands indicate the cleaved fragments of DNA from the sgRNA/Cas9 complex. Controls are shown on the far-right side. 2kb ladder was used for fragment size identification.

The spacer sgRNA portion of CRISPR/Cas9, a fundamental component of the bacterial immune system, evolved to interact with viral DNA of approximately 17–20 bases in order to be the most efficient and selective[6]. Application of CRISPR/Cas9 gene editing to mammalian systems will likely require a process of optimization for every intended target cleavage site. In the present study, sgRNAs of differing lengths designed towards three varied PAM sites were evaluated to determine if extension of sgRNA spacer length enhanced or hindered specific cleavage of the GGTA1 gene. Before sgRNA could be tested and analyzed for native and off-target DNA template cleavage, sgRNA were screened to determine potential for facilitating CRISPR/Cas9 mediated cleavage. Guides were tested against the porcine GGTA1 gene that was asymmetrically PCR amplified at the targeted PAM sites. Cleavage assays demonstrated that all 10 sgRNA evaluated in this study were able to successfully form RNPs and cleave the GGTA1 DNA template (Fig 2). Data presented supported further evaluation of the specificity and efficiency of each sgRNA by screening against designed off-target DNA templates and comparing observed OTC to that of the native GGTA1 DNA template cleavage.

### Off-target and native DNA template cleavage

**Fig 3:**
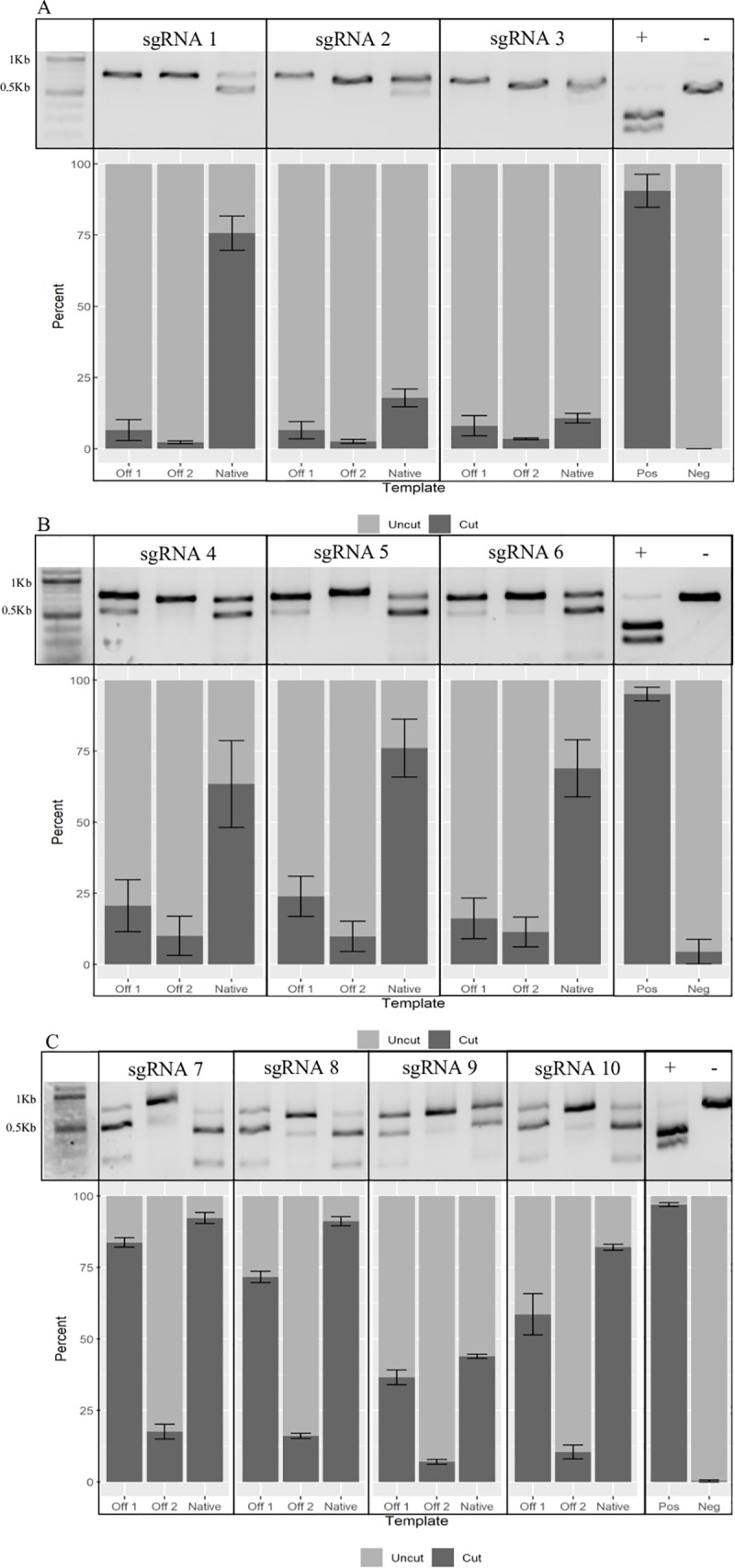
sgRNA/Cas9 cleavage of off-target and native DNA templates. Cleavage efficiency of sgRNA with varied length for each target DNA sequence and PAM site was determined by densitometric analysis of cleavage products: A) PAM1 B) PAM2 C) PAM3. The average of three cleavage reactions for each PAM / sgRNA was used to generate a representative bar graph for each sgRNA and DNA template. Controls are include. Standard error of the mean is shown as error bars (+/-).

Following confirmation of successful RNP formation and cleavage of the genomic GGTA1 DNA template for all sgRNA evaluated, regardless of length, the sgRNA was tested for cleavage efficiency and specificity of the native GGTA1 DNA template over the off-target templates for each PAM site using the designed synthetic dsDNA. The *in vitro* cleavage assays were performed in triplicate and the resulting cleavage products were run on gels and densitometry was performed to determine the percent cleavage of each DNA template. Results from the assays were averaged and the data is shown in Table 3 and Fig 3A–3C.

**Table 3:** Cleavage data averaged from cleavage assays products ran in triplicate (n = 3) and analyzed by densitometry.

Guide ID	Template ID	Avg.Uncleaved Template (%)	Avg. Cleaved Template (%)	Standard Error of the mean (+/-)
1	Off Target 1	93.47	6.53	3.70
1	Off Target 2	97.71	2.29	0.51
1	Native	24.28	75.72	6.03
2	Off Target 1	93.50	6.50	3.08
2	Off Target 2	97.43	2.57	0.66
2	Native	82.14	17.86	3.14
3	Off Target 1	91.92	8.08	3.55
3	Off Target 2	96.54	3.46	0.32
3	Native	89.25	10.75	1.69
Pos	Pos	9.46	90.54	5.75
Neg	Neg	100.00	0.00	0.00
4	Off Target 1	79.32	20.68	9.15
4	Off Target 2	89.93	10.07	6.91
4	Native	36.53	63.47	15.24
5	Off Target 1	76.03	23.97	7.06
5	Off Target 2	90.13	9.87	5.35
5	Native	23.88	76.12	10.20
6	Off Target 1	83.81	16.19	7.19
6	Off Target 2	88.61	11.39	5.25
6	Native	31.02	68.98	10.04
Pos	Pos	4.87	95.13	2.40
Neg	Neg	95.50	4.50	4.30
7	Off Target 1	16.26	83.74	1.63
7	Off Target 2	82.43	17.57	2.57
7	Native	7.72	92.28	1.94
8	Off Target 1	28.31	71.69	2.01
8	Off Target 2	83.89	16.11	0.90
8	Native	8.82	91.18	1.60
9	Off Target 1	63.39	36.61	2.60
9	Off Target 2	92.99	7.01	0.86
9	Native	56.08	43.92	0.66
10	Off Target 1	41.43	58.57	7.19
10	Off Target 2	89.54	10.46	2.43
10	Native	17.94	82.06	1.08
Pos	Pos	3.06	96.94	0.66
Neg	Neg	99.88	0.12	0.34

Standard error is shown (+/-).

Results show sgRNA directed towards PAM1 demonstrated 75.72% cleavage efficiency for 20 bp (sgRNA1), 17.86% for 30 bp (sgRNA2), and 10.75% for 40 bp (sgRNA3), with a difference in cleavage efficiency of 64.97% between the longest and shortest sgRNA evaluated (Fig 3A, Table 3). No significant differences were seen between the three guides at the PAM2 cut site; a cleavage efficiency of 63.47% was seen for 20 bp (sgRNA4), 76.12% for 30 bp (sgRNA5), and 68.98% for 40 bp (sgRNA6) (Fig 3B, Table 3). In contrast, however, at the PAM3 cut site, cleavage efficiency of 92.28% was seen for 19 bp (sgRNA7), 91.18% for 30 bp (sgRNA8), 43.92% for 40 bp (sgRNA9), and 82.06% for 53 bp (sgRNA10) (Fig 3C, Table 3). For the PAM3 cut site, a relative difference in cleavage efficiency of 48.36% was seen between 19 bp (sgRNA7) and 40 bp (sgRNA9), however, only a 10.22% difference was seen between the 19 bp (sgRNA7) and 53 bp (sgRNA10) (Fig 3C, Table 3).

To optimize CRISPR/Cas9 gene editing, maximal cleavage efficiency and specificity for the target gene are critical. Thus, it is crucial to evaluate the relationship between guide length and the potential of off-target binding sites in the context of GGTA1 KO in porcine donors for xenotransplantation.

Cleavage efficiency of off-target DNA templates was analyzed to determine specificity of the extended guides by comparing them to standard 19–20 bp length sgRNA spacers. Results of this study demonstrate that, depending on the PAM site and sgRNA spacer length used, an increased likelihood of OTC was seen depending on the targeted PAM site. For PAM1, no significant differences were found for either off-target template; sgRNA1 (20 bp) had OTC of 6.53% and 2.29%, sgRNA2 (30 bp) had an OTC of 6.50% and 2.57%, and sgRNA3 (40 bp) had an OTC of 8.08% and 3.46%, for off-target templates 1 and 2, respectively (Fig 3A, Table 3). Similarly, for the PAM2 cut site, no statistically significant differences were found for any of the sgRNA evaluated, regardless of sgRNA length; sgRNA4 (20 bp) had OTC of 20.68% and 10.07%, sgRNA5 (30 bp) had OTC of 23.97% and 9.87%, and sgRNA6 (40 bp) had OTC of 16.19% and 11.39%, for off-target templates 1 and 2, respectively (Fig 3B, Table 3).

Unlike PAM1 and PAM2, results for the PAM3 cut site were more variable. Significant differences between each of the four sgRNA tested were seen for off-target template 1; sgRNA7 (19 bp) cut 83.74%, sgRNA8 (30 bp) cut 71.69%, sgRNA9 (40 bp) cut 36.61%, and sgRNA10 (53 bp) cut 58.57% of the template (Fig 3C, Table 3). Moreover, sgRNA7 (19 bp) was the least specific for native template, with only an 8.54% difference between native and off-target template 1, while the 53 bp sgRNA10 was shown to be the most specific, with a difference of 23.49% between off-target template 1 and native template. For off-target template 2, only sgRNA9 was significantly different from other guides tested; sgRNA7 cut 17.57%, sgRNA8 cut 16.11%, sgRNA9 cut 7.01%, and sgRNA10 cut 10.46% for off-target template 2 (Fig 3C, Table 3).

### Screening GTKO cell lines and piglet DNA for off-target mutations

As shown above the CRISPR/Cas9 system has the capability to bind to OTB and create double stranded DNA breaks. To determine if the sgRNAs that were designed in this experiment were actually producing OTM in cell lines and in a pig, PCR primers were designed to screen both GTKO cell lines and a GTKO pig for OTMs. In total we selected 6 different cell lines and 1 piglet to screen for GGTA1 OTMs. The GGTA1 gene was targeted for KO using sgRNA7. As our results showed in (Fig 3C and Table 3) this sgRNA had the highest rate of *in vitro* OTC, and was the most likely sgRNA to produce OTM in transfected cells. After transfect cells were sorted (BD Bioscience, FACS Melody) and confirmed to be GTKO prior to being screened for OTMs. In total we screened for mutations at 8 different OTBs. The isolated DNA underwent PCR at the 8 OTBs (S1 Table) and was then sent for Sanger sequencing. Sanger sequencing revealed that none of the cell lines or the piglet had any detectable at OTMs (S1 Fig).

## Discussion

The use of the CRISPR/Cas9 gene editing system for the selective generation of indels in somatic cells creates an opportunity to study cell function *in vitro*, as well as to produce GE cells and new animal models. In many cases, sgRNA are designed with the conventional 20 bp spacer sequence and used to transfect and collect cells displaying a modified phenotype, without first exploring whether that sgRNA is, in fact, the most efficient and specific sgRNA for a given target gene. Low efficiency can dramatically increase the cost of such experiments, and even a marginal lack of specificity can create unwanted OTM. We sought to develop a better understanding of how sgRNA spacer length can affect target specificity by varying sgRNA length in an *in vitro* DNA cleavage system to evaluate efficiency, specificity, and the likelihood of binding to off-target sites using the GGTA1 gene as a model target. GE cell lines and GE animals that were created using sgRNA7 were then screened for OTM to determine if mutations can be detected in viable cell lines or in live animals.

sgRNA were designed targeting exon 1 of the GGTA1 gene. ZiFit Targeter software was used to identify three proximal PAM sites within the first 55 nt following the start codon (Methionine) of the exon. Targeting exon 1 and close proximity to the start codon may provide the most efficient indels, minimizing the creation of truncated proteins, resulting in the creation of healthier GE somatic cells. Guides for the PAM1 site, located 8 bp after the start codon, were designed with lengths of 20, 30, and 40 bp, all with binding regions outside of exon 1 of the GGTA1 gene. Similarly, guides for the PAM2 site, located 16 bp after the start codon, were designed to have lengths of 20, 30, and 40 bp. All three guides for the PAM2 site have DNA binding regions extending into the non-coding region of the GGTA1 gene. Guides for the PAM3 site, positioned 49 bp after the start codon, were designed with spacer lengths of 19, 30, 40, and 53 bp. For PAM3, the designed sgRNA had DNA binding regions exclusively located within the exon 1 region, with the exception of the longest guide, sgRNA10 (53 bp), which extended 1 bp outside of the exon.

Following sgRNA design, it was necessary to validate that all guides were capable of forming RNPs with the Cas9 protein and cleaving GGTA1 DNA sequence amplified from genomic porcine DNA. In this case, PAM sites were asymmetrically located within the genomic sequence such that cleavage resulted in fragments of different sizes, allowing for verification of successful cleavage (Fig 2). Results confirmed that all sgRNAs were able to successfully form RNPs and cleave GGTA1 DNA sequences, regardless of sgRNA length. Subsequently, two synthetic dsDNA templates were created that represented the two most likely off-target binding sites found in the porcine genome for each of the three PAM sites, designated as Off-Target #1 and #2 for each PAM site (Table 2).

A cell-free *in vitro* system was used to exclude the impact of guide degradation and transfection loss to the experiments. Results of this experiment are limited in their scope, for it is unknown whether the Cas9 enzyme will protect extended sgRNA from degradation in target cells, or how the sgRNA/Cas9 complex will perform in a cellular setting.

To ensure a consistent source of off-target template free of replication or PCR based errors, synthetic dsDNA representing a 600 bp region of the GGTA1 gene were asymmetrically constructed around the chosen PAM sites. In order to mimic the most likely potential off-target sequences, only those bases that differed from the native sequence within the 20 bp binding spacer region were modified. The PAM1 Off-Target #1 sequence (accession CU914429.7) represents an un-annotated region of the X chromosome in the porcine genome. Much of the porcine genome is un-annotated, therefore, a search of the human genome was conducted to determine whether the chosen sequence aligned with any human genes or transcripts. This sequence aligned with an uncharacterized transcript of non-coding RNA in the human genome. The PAM1 Off-Target #2 sequence matched un-annotated sequences on Chromosome X and 7 (accessions FP016194.8 and CT841554.8) of the porcine genome. In addition, the PAM1 Off-Target #2 sequence matched to the glycoprotein, α-galactosyltransferase 1 pseudogene (GGTA1P), in humans. Based on this analysis, the impact of potential off-target mutations remains unclear.

PAM2 Off-Target #1 sequence (accession FP102187.6) referenced to a sequence found on Chromosome X in the porcine genome. There are currently no known characterized transcripts in the porcine genome, however, the sequence aligned with the endoplasmic reticulum aminopeptidase 1 (ERAP1) in humans. ERAP1 has been shown to be involved in the processing and trimming of peptides in the endoplasmic reticulum for presentation on MHC I molecules; KO of ERAP1 results in a reduction in the ability of the host to mount a proper immune response[16]. The PAM2 Off-Target #2 template (accession XM_021073251.1) is predicted to encode for an mRNA transcript for aminoadipate aminotransferase (AADAT). AADAT is an enzyme found primarily in the brain and known to catalyze the transamination of kynurenine to kynurenic acid; AADAT is also responsible for the transamination of aminoapidate to alpha-oxoadipate[17]. Mutations to either of these enzymes could have significant impacts on the health of any cell, and piglets produced from GE cells with these mutations would be unlikely to survive a full-term pregnancy.

Finally, the PAM3 Off-Target #1 sequence (accession XM_013993817.2) matches a series of GGTA1P transcript variants in the porcine genome. The PAM3 Off-Target #2 template (accession AB206998.1) codes for the agouti signaling protein and S-adenosylhomocysteine hydrolase; genetic mutations found in this gene have been linked to a disorder of methionine metabolism in humans, characterized by psycho-motor and metabolic dysfunction[18]. Without medical intervention, it unlikely that pigs born with this mutation could survive neo-natal development.

The use of synthetic dsDNA to make the mutated templates provides a consistent and validated source of DNA free of PCR based errors. With current methods of analysis, the use of dsDNA is necessary in order to consistently measure the effect of varied sgRNA length *in vitro*, without the risk of any unintentional PCR based mutations confounding the results. Importantly, however, synthetic dsDNA lacks any potential epigenetic modification to DNA naturally occurring in a cell that could impact CRISPR/Cas9 mediated cleavage. Following design of six total off-target sequences, determination of sgRNA specificity was necessary to evaluate whether extended sgRNA length results in increased specificity for the native template when compared to off-target templates. Evaluation of potential off-target binding sites prior to the transfection of GE cells for the production of animal donors is crucial to determine whether target genes, if knocked out, could negatively impact cells *in vitro* or otherwise affect fetus development.

Multiple studies have proposed the concept of modifying the length of the sgRNA spacer component for improved CRISPR/Cas9 specificity and reduced OTM[19–22]. The use of truncated guide RNA (tru-gRNA) and tru-gRNA guided nucleases have been shown to reduce OTM, while maintaining efficiencies similar to those obtained with conventional 20 bp length sgRNA [19]. In 2014, Fu et al. reduced sgRNA spacer length from 20 nt to 17 nt without observing significant changes in activity and OTM were substantially reduced[19]. In contrast, when tru-gRNA length was decreased to 16 nt, activity either decreased substantially or ceased altogether[19]. Based on these results, Fu et al. speculated that sgRNA with shorter spacer regions are less likely to bind to mismatched sequences, resulting in increased specificity[19].

In this study, results of cleavage with sgRNA for PAM1 were especially interesting because a large decrease in efficiency was observed with extension of sgRNA length (Fig 3A, Table 3). The data showed that there was a 64.97% decrease in native template cleavage as the sgRNA was increased in length. (Fig 3A, Table 3). In addition, no statistically significant differences in specificity were observed; regardless of length, OTC was within 2%-8% for all three sgRNA used at the PAM1 site (Fig 3A, Table 3). A possible explanation for this observed decrease in DNA cleavage is that, as guides extended further into the non-exon region of the GGTA1 gene, sgRNA encountered DNA sequences containing high amounts of cytosine and thymine repeats, which may have inhibited the binding of the sgRNA to the DNA template. Overall, at the PAM1 site, the standard 20 bp length sgRNA was determined to be the most efficient at cleaving the GGTA1 DNA template; furthermore, no observed differences in the specificity of the different guides for the native versus off-target template were seen (Fig 3A, Table 3).

When examining the sgRNA for the PAM2 site, a different trend among the DNA template cleavage was observed, despite PAM2 being only 5 bp further into the exon of the gene, relative to PAM1. While no statistically significant differences were found between the three sgRNA at the PAM2 site, overall, longer guides showed higher cleavage efficiencies when compared to sgRNA4 (20 bp) (Fig 3B, Table 3). Furthermore, the observed increase in cleavage efficiency did not correlate to a significant increase in specificity for the native template. Results of this study demonstrate the importance of sgRNA screening and the potential impact of guide length on both efficiency and specificity, depending on the PAM site selected and distance from the start codon for the GGTA1 gene.

The final target site for the GGTA1 gene that was investigated in this study was the PAM3 site, positioned the furthest into the exon of the three PAM sites evaluated in this study, at 49 bp after the start codon. Results for the PAM3 site were unique; the PAM3 site gave the highest cleavage efficiencies for native template seen in this experiment and also the highest occurrence of OTC. Cleavage efficiency for the native template ranged from 92.28% for sgRNA7 (19 bp) to 43.92% for sgRNA9 (40 bp) (Fig 3C, Table 3). No significant differences were found between the 20, 30, and 53 bp sgRNA for native template cleavage, supporting the conclusion that extending the guide length at PAM3 can, at least, maintain target DNA template cleavage efficiencies. Furthermore, the most interesting results were seen at PAM3 when evaluating the impact of guide length extension on specificity.

This study demonstrates that while the shortest guide (sgRNA7) had the highest native GGTA1 template cleavage efficiency across all PAM sites, sgRNA7 was also associated with the most OTC of any of the guides evaluated in this study. Although shorter spacer length tended to be correlated with higher efficiency at each of the PAM sites, in this case, truncated sgRNA were also associated with increased OTC.

The findings shown here are supported by previous studies, which have demonstrated that sgRNA spacer length extension can results in decreased efficiency[19,20,22,23]. Ran et al. compared the cleavage specificity in human cells for sgRNA with the standard 20 bp spacer to an extended sgRNA with a 30 bp spacer, however, no appreciable differences in activity were observed[20]. In contrast, Hsu et al. compared the efficiency of CRISPR gene editing using trans-activating CRISPR RNA (tracrRNA) modified with tails of either +67 or +85 nt and concluded that increasing the length of tracrRNA resulted in increased cleavage at the target gene site[22]. Moreover, in 2016, Zhang et al. concluded that, in stem cells, the use of tru-gRNA with 17 nt spacer components resulted in decreased OTM, as well as decreased KO efficiency, making the 20 bp sequence sgRNA preferable for its increased efficiency[21]. In contrast to what others have found[19,20,22,23], results of the our research align with the conclusions of Dang et al., who found that extending the sgRNA spacer length improved or maintained efficiency when compared to the conventional 20 bp length guide spacer[24].

It was initially predicted that by extending the length of the sgRNA, the specificity of the system would be improved. This was attributed to an increased number of available RNA-DNA binding sites, which are required for the sgRNA to successfully bind to the DNA target, thus, decreasing the potential for OTM and potentially increasing the efficiency of CRISPR/Cas9. Using current technology, we have been able to successfully design guides that produce greater than 90% indel formations at their target gene sites (unpublished data). Whether it was possible to improve on such high efficiency levels was unknown, however, the intention of this study was to maintain the efficiency of the current CRISPR/Cas9 gene editing system for the GGTA1 gene while increasing the specificity for GGTA1 by extending guide length to decrease the potential for off-target cleavage. The data supports the conclusion that extended guide length improves or maintains cleavage efficiency for the GGTA1 gene sequence and reduces off-target cleavage, although the extent of this improvement is clearly dependent on PAM site.

The present study differed from previous work by evaluating the impact of extended spacer length and the specificity of the Cas9 mediated cleavage for the native DNA template. Results show that no significant changes in specificity were found for the native template versus the off-target template for PAM1 and PAM2 sites. In contrast, for the PAM3 site, as guide length was extended, a corresponding decrease in OTC was observed. Significant variation between native and off-target template cleavage for the PAM3 site was found, regardless of sgRNA spacer length. This finding might further shed light on the importance of PAM site selection when designing guides for selected gene KO using the CRISPR/Cas9 gene editing system. Differences were observed in the native and off-target template cleavage efficiency as distance between start codon and PAM site was increased in the GGTA1 gene. Therefore, PAM site selection and sgRNA length are critical factors that are necessary to evaluate in order to reduce the potential of OTM when producing GE cells and animals for xenotransplantation.

While it is evident by the results of the *in vitro* studies that OTMs are possible when using the CRISPR/Cas9 system, it is important to understand if OTM can be found in GTKO cells lines or piglets produced from those cells. We screened DNA from both sources for 8 different OTMs. While it is unlikely that OTM would occur at OTB that do not include a PAM site, we screened for OTM at OTB that did not include a PAM site, in order to include as many off-target sites as possible. We found that by screening DNA from both GTKO cell lines and a GTKO pig that there were no detectable OTM in the 8 sites that we evaluated (S1 Fig). This could be because the OTM occurring in these cells are lethal and cells do not survive in culture after the engineering process and therefore cell lines with OTMs cannot be established and no piglets will be born with OTM. A further analysis of the 8 OTB that were screened showed that a majority of the OTB that were identified were genes that were important for cell survival, and unlikely to produce a viable cell after mutations occur.

This study highlights a strategy for minimizing off-target events when employing CRISPR/Cas9 for potential use in the field of xenotransplantation for GTKO. Moreover, analysis of potential OTC may be critical to improving efficiency while maintaining the integrity of cells intended for use in functional screening of mutated genes in cells or the production of GE animals. OTM occurring in cells used to create GE animals could lead to the development of animals with unknown gene modifications with the potential to affect both the donor animal and recipients of xenografts. No OTM were found in any of the GTKO cell lines or the pig we screened leading us to believe that if OTM occur in critical genes those cells may not develop and survive in culture. Findings presented here underscore the importance of evaluating and screening guide efficiency and specificity to produce GTKO GE cells, as well as the importance of PAM site selection and its role in optimizing both efficiency and specificity of the CRISPR/Cas9 gene editing system.

## Supporting information

S1 Fig Sanger sequencing data for OTM at eight OTB. (A) All cell lines and the piglet used were confirmed GTKO and the GGTA1 sequence is shown compared to the wild type GGTA1 sequence. (B-I) shows sequence data for all 8 OTB and are compared to a wild type sequence of the OTB to determine if OTM occurred. No OTM were seen for any of the OTB screened.
(TIF)

S1 Raw Figure Unmodified images of SDS page gels used to create Fig 2 and Fig 3A–3C. (PDF)

S1 Table Off-target binding site sequences that were used for off-target mutation screening in GTKO cell lines and GTKO piglet. OTB 1–3 included PAM sites, OTB 4–8 did not have a PAM site associated with off-target sequence. Mismatched nts are bolded and underlined.
(DOCX)

S2 Table Primers used for PCR and Sanger sequencing of GGTA1 and OTBs in wild type and GTKO cell lines and GTKO piglet. (DOCX)

## Abbreviations

α-Gal: Galactose-α-1,3-galactose
AADAT: aminoadipate aminotransferase
bp: base pair
BLAST: basic local alignment search tool
β4GalNT2 β-1: 4N-acetylgalactosaminyltransferase
Cas9: CRISPR, associated protein 9
CMAH: cytidine monophosphate-N-acetylneuraminic acid hydroxylase
CRISPR: clustered randomly interspersed short palindromic repeats
GGTA1: α-1,3-galactosyltransferase
GGTA1P: α-galactosyltransferase 1 pseudogene
GTKO: α-Gal KO
IgG: immunoglobulin G
IgM: immunoglobulin M
KO: knockout
Nt: nucleotide
OTM: off-target mutation
OTC: off-target cleavage
PAM: protospacer adjacent motif
PBMC: peripheral blood mononuclear cells
PFF: porcine fetal fibroblast
RNP: ribonucleoprotein
sgRNA: single-guide RNA
TALENs: transcription activator-like effector nucleases
tracrRNA: trans-activating CRISPR RNA
tru-gRNA: truncated RNA
WT: wild type
ZFNs: zinc-finger nucleases

## References

[1] Naeimi Kararoudi M, Hejazi SS, Elmas E, Hellström M, Naeimi Kararoudi M, Padma AM, et al Clustered regularly interspaced short palindromic repeats/Cas9 gene editing technique in xenotransplantation. Front Immunol [Internet]. 2018;9(1711):1–7. Available from: https://www.frontiersin.org/article/10.3389/fimmu.2018.01711/full 30233563 10.3389/fimmu.2018.01711 PMC6134075

[2] Sato M, Miyoshi K, Nagao Y, Nishi Y, Ohtsuka M, Nakamura S, et al The combinational use of CRISPR/Cas9-based gene editing and targeted toxin technology enables efficient biallelic knockout of the α-1,3- galactosyltransferase gene in porcine embryonic fibroblasts. Xenotransplantation [Internet]. 2014;21(3):291–300. Available from: 10.1111/xen.12089 24919525

[3] Burlak C, Paris LL, Lutz AJ, Sidner RA, Estrada J, Li P, et al Reduced binding of human antibodies to cells from GGTA1/CMAH knockout pigs. Am J Transpl [Internet]. 2014;14(8):1895–900. Available from: https://www.ncbi.nlm.nih.gov/pmc/articles/PMC4366649/10.1111/ajt.12744 PMC4366649 24909344

[4] Estrada J, Martens G, Li P, Adams A, Newell K, Ford M, et al Evaluation of human and nonhuman primate antibody binding to pig cells lacking GGTA1/CMAH/β4GalNT2 genes. Xenotransplantation [Internet]. 2015;22(3):194–202. Available from: 10.1111/xen.12161 25728481 PMC4464961

[5] Jinek M, East A, Cheng A, Lin S, Ma E, Doudna J. RNA-programmed genome editing in human cells. Elife [Internet]. 2013;2:1–9. Available from: 10.7554/eLife.00471 PMC3557905 23386978

[6] Jiang F, Doudna JA. The structural biology of CRISPR-Cas systems. Curr Opin Struct Biol [Internet]. 2015;30:100–11. Available from: 10.1016/j.sbi.2015.02.002 25723899 PMC4417044

[7] Jiang F, Doudna JA. CRISPR–Cas9 structures and mechanisms. Annu Rev Biophys [Internet]. 2017;46:505–29. Available from: 10.1146/annurev-biophys-062215-010822 28375731

[8] Gaj T. ZFN, TALEN and CRISPR/Cas based methods for genome engineering. Trends Biotechnol [Internet]. 2014;31(7):397–405. Available from: https://www.ncbi.nlm.nih.gov/pubmed/23664777 10.1016/j.tibtech.2013.04.004 PMC3694601 23664777

[9] Peng R, Lin G, Li J. Potential pitfalls of CRISPR/Cas9-mediated genome editing. FEBS J [Internet]. 2016;283(7):1218–31. Available from: 10.1111/febs.13586 26535798

[10] Fu Y, Foden J a, a C, Maeder ML, Reyon D, Joung K, et al High frequency off-target mutagenesis induced by CRISPR-Cas nucleases in human cells. Nat Biotechnol [Internet]. 2013;31(9):822–6. Available from: 10.1038/nbt.2623 23792628 PMC3773023

[11] Yang H, Wu Z. Genome Editing of Pigs for Agriculture and Biomedicine. Front Genet [Internet]. 2018;9(September):360 Available from: https://www.frontiersin.org/article/10.3389/fgene.2018.00360/full 30233645 10.3389/fgene.2018.00360 PMC6131568

[12] Sander JD, Zaback P, Joung JK, Voytas DF. Zinc Finger Targeter (ZiFiT): an engineered zinc finger/target site design tool. Nucleic Acids Res [Internet]. 2007;35:599–605. Available from: https://www.ncbi.nlm.nih.gov/pubmed/20435679 10.1093/nar/gkm349 PMC1933188 17526515

[13] Sander JD, Maeder ML, Reyon D, Voytas DF, Joung JK, Dobbs D. ZiFiT (zinc finger targeter): an updated zinc finger engineering tool. Nucleic Acids Res [Internet]. 2010;38:462–8. Available from: https://www.ncbi.nlm.nih.gov/pubmed/20435679 10.1093/nar/gkq319 PMC2896148 20435679

[14] Takara. Guide-it TM sgRNA in vitro transcription and screening systems user manual [Internet]. 2017. Available from: takarabio.com

[15] Bae S, Park J, Kim J-S. Cas-OFFinder: a fast and versatile algorithm that searches for potential off-target sites of Cas9 RNA-guided endonucleases. Bioinformatics. 2014;30(10):1473–5. 10.1093/bioinformatics/btu048 24463181 PMC4016707

[16] York IA, Brehm MA, Zendzian S, Towne CF, Rock KL. Endoplasmic reticulum aminopeptidase 1 (ERAP1) trims MHC class I-presented peptides in vivo and plays an important role in immunodominance. Proc Natl Acad Sci. 2006;103(24):9202–7. 10.1073/pnas.0603095103 16754858 PMC1482590

[17] Han Q, Cai T, Tagle DA, Robinson H, Li J. Substrate specificity and structure of human aminoapidate aminotransferase/kynurenine aminotransferase II. Biosci Rep [Internet]. 2008;28(4):205–15. Available from: https://www.ncbi.nlm.nih.gov/pubmed/18620547 10.1042/BSR20080085 18620547 PMC2559858

[18] Barić I, Fumic K, Glenn B, Schulze A, Finkelstein JD, James SJ, et al S-adenosylhomocysteine hydrolase deficiency in a human: A genetic disorder of methionine metabolism. Proc Natl Acad Sci [Internet]. 2004;101(12):4234–9. Available from: https://www.pnas.org/content/101/12/4234 10.1073/pnas.0400658101 15024124 PMC384724

[19] Fu Y, Sander JD, Reyon D, Cascio VM, Joung JK, Unit P, et al Improving CRISPR-Cas nuclease specificity using truncated guide RNAs. Nat Biotechnol [Internet]. 2014;32(3):279–84. Available from: https://www.ncbi.nlm.nih.gov/pubmed/24463574 10.1038/nbt.2808 24463574 PMC3988262

[20] Ran FA, Hsu PD, Lin C, Gootenberg JS, Trevino A, Scott DA, et al Double nicking by RNA-guided CRISPR Cas9 for enhanced genome editing specificity. Cell [Internet]. 2014;154(6):1380–9. Available from: https://www.cell.com/abstract/S0092-8674(13)01015-5 10.1016/j.cell.2013.08.021 PMC3856256 23992846

[21] Zhang JP, Li XL, Neises A, Chen W, Hu LP, Ji GZ, et al Different effects of sgRNA length on CRISPR-mediated gene knockout efficiency. Sci Rep [Internet]. 2016;6:1–10. Available from: 10.1038/s41598-016-0001-8 27338021 PMC4919781

[22] Hsu PD, Scott DA, Weinstein JA, Ran FA, Konermann S, Agarwala V, et al DNA targeting specificity of RNA-guided Cas9 nucleases. Nat Biotechnol [Internet]. 2013;31(9):827–32. Available from: 10.1038/nbt.2647 23873081 PMC3969858

[23] Cho SW, Kim S, Kim Y, Kweon J, Kim HS, Bae S, et al Analysis of off-target effects of CRISPR/Cas-derived RNA-guided endonucleases and nickases. Genome Res [Internet]. 2014;24:132–41. Available from: 10.1101/gr.162339.113 24253446 PMC3875854

[24] Dang Y, Jia G, Choi J, Ma H, Anaya E, Ye C, et al Optimizing sgRNA structure to improve CRISPR-Cas9 knockout efficiency. Genome Biol [Internet]. 2015;16(280):1–10. Available from: 10.1186/s13059-015-0846-3 26671237 PMC4699467

