# Supplementary figures and images for: Optimizing sgRNA length to improve target specificity and efficiency for the GGTA1 gene using the CRISPR/Cas9 gene editing system
Source: PLoS One. 2019 Dec 10;14(12):e0226107. doi: 10.1371/journal.pone.0226107 (PMC6903732; doi:10.1371/journal.pone.0226107)

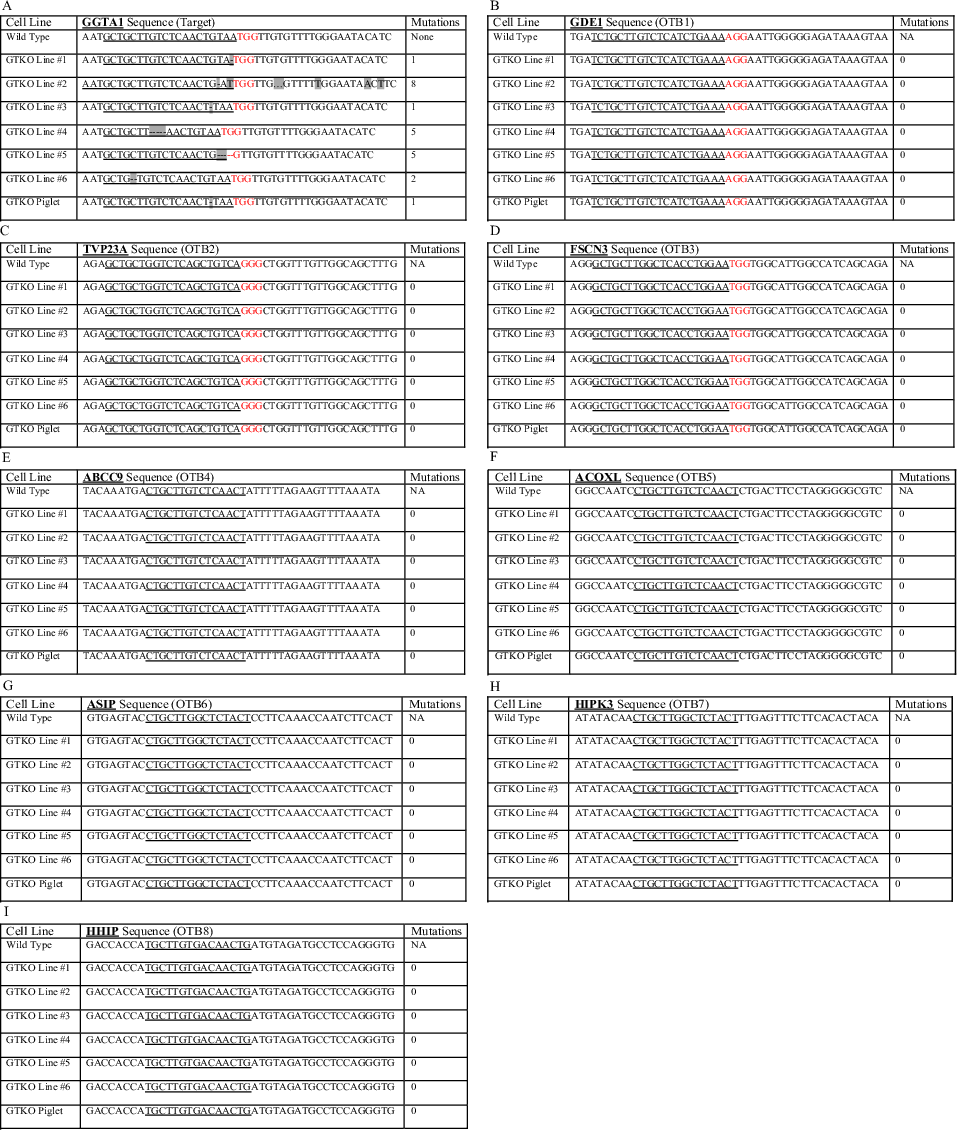

Supplement: S1 Fig — (A) All cell lines and the piglet used were confirmed GTKO and the GGTA1 sequence is shown compared to the wild type GGTA1 sequence. (B-I) shows sequence data for all 8 OTB and are compared to a wild type sequence of the OTB to determine if OTM occurred. No OTM were seen for any of the OTB screened. (TIF) [file pone.0226107.s001.tif]

**Fig 2 Raw Image**

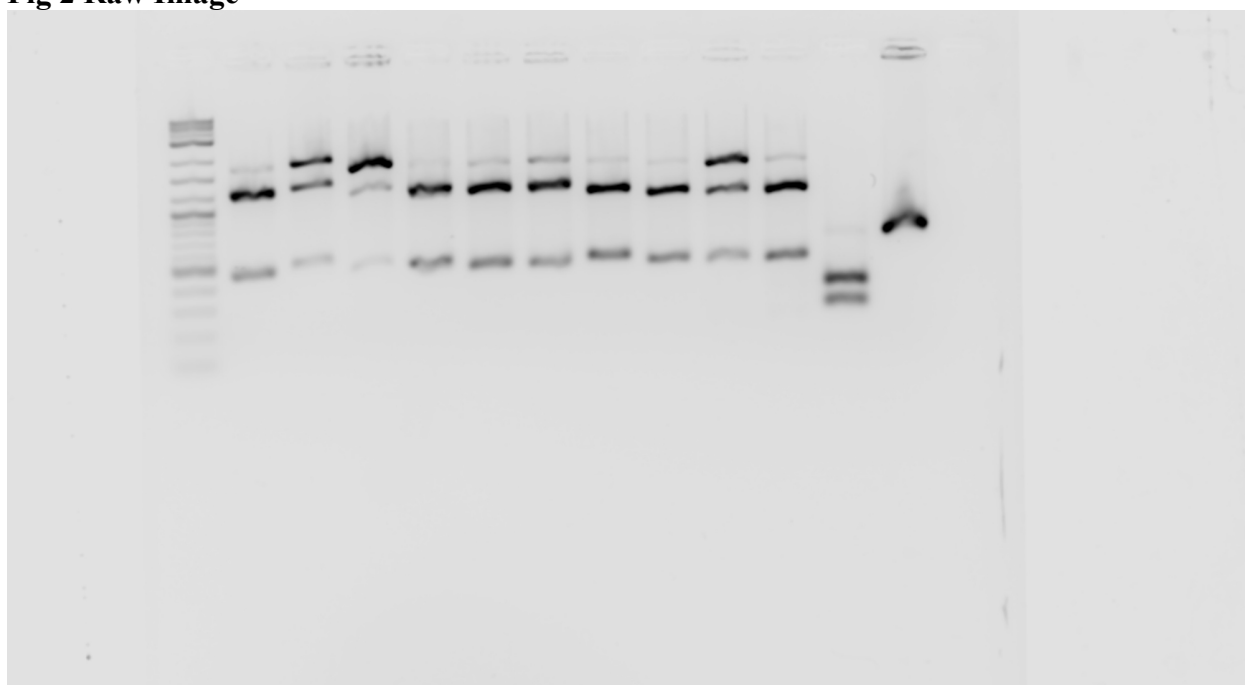

**Fig 3 Raw Image**

**A**

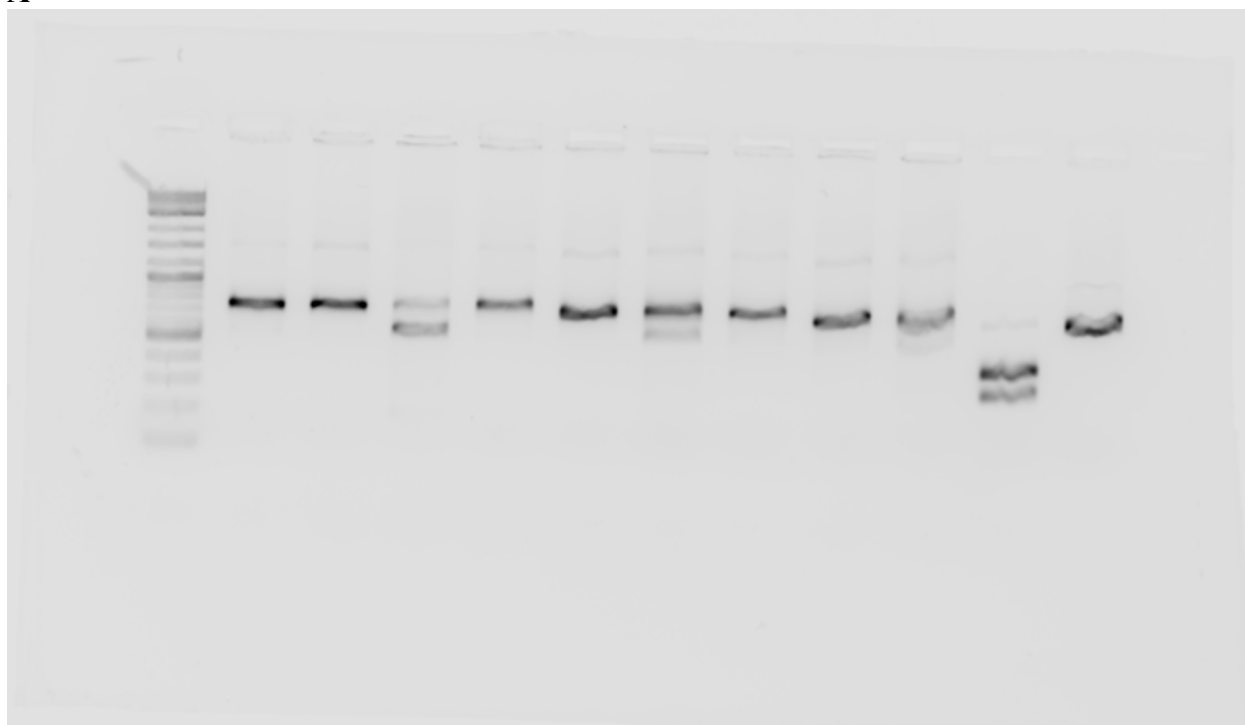

**B**

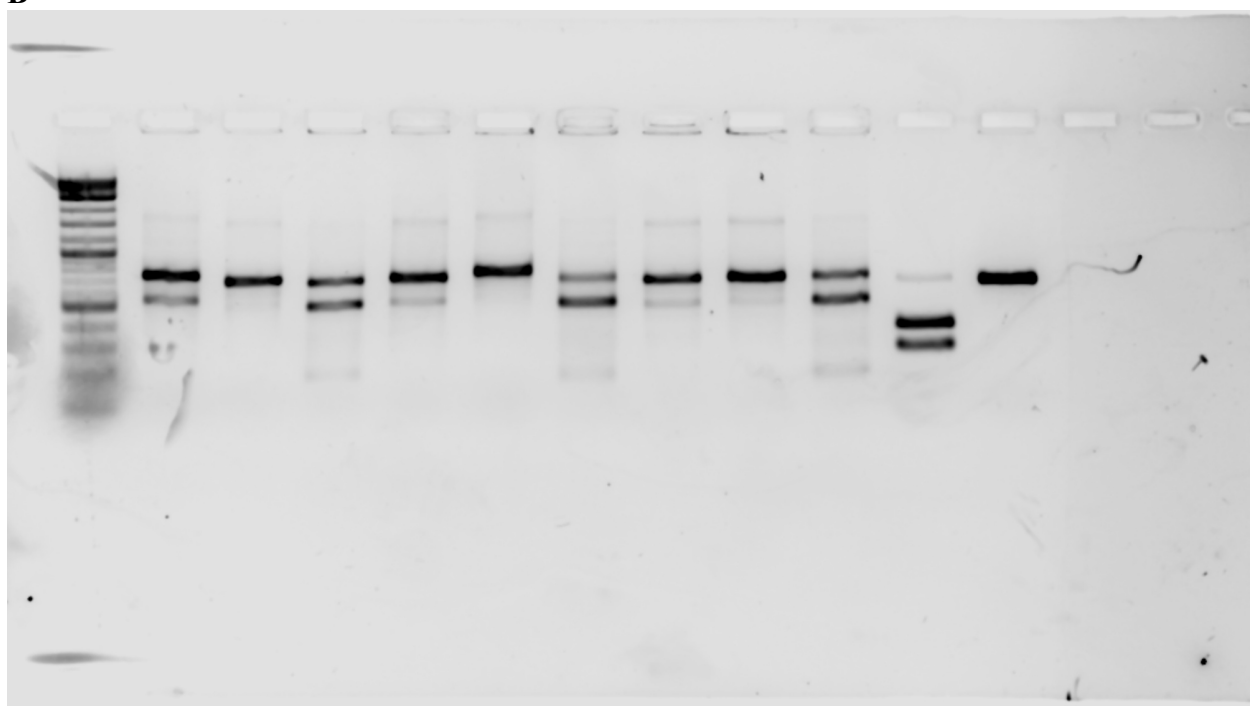

**C**

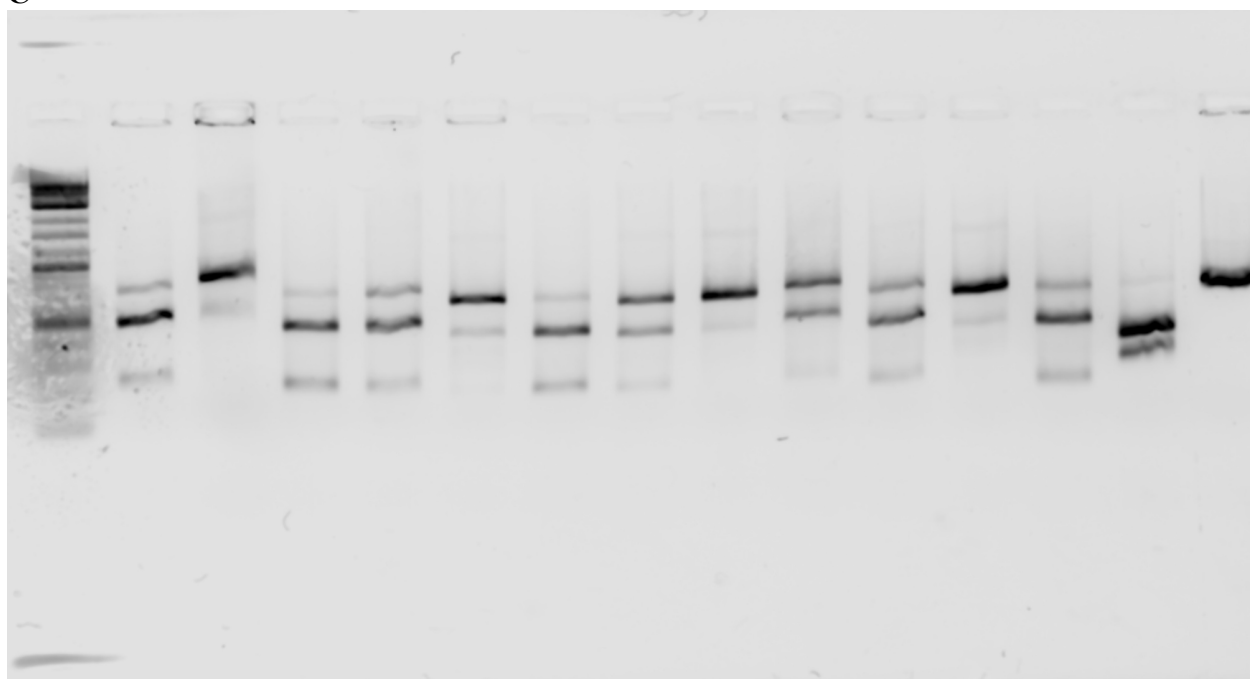

Supplement: S1 Raw Figure — (PDF) [file pone.0226107.s002.pdf]
