# Supplementary material for: Optimizing sgRNA length to improve target specificity and efficiency for the GGTA1 gene using the CRISPR/Cas9 gene editing system
Source: PLoS One. 2019 Dec 10;14(12):e0226107. doi: 10.1371/journal.pone.0226107 (PMC6903732; doi:10.1371/journal.pone.0226107)
Supplement: S1 Table — OTB 1–3 included PAM sites, OTB 4–8 did not have a PAM site associated with off-target sequence. Mismatched nts are bolded and underlined. (DOCX) [file pone.0226107.s003.docx]

**S1 Table.**

| Template ID | DNA Sequence | Orientation | Accession no. | Gene ID |
| --- | --- | --- | --- | --- |
| Native (GGTA1) | GCTGCTTGTCTCAACTGTAA*NGG | + | NC_010443.5 | 396733 |
| OTB1 (GDE1) | **T**CTGCTTGTCTCA**T**CTG**A**AA*NGG | - | NC_010445.4 | 100624000 |
| OTB2 (TVP23A) | GCTGCT**G**GTCTCA**G**CTGT**C**A*NGG | + | NC_010445.3 | 100620244 |
| OTB3 (FSCN3) | GCTGCTTG**G**CTCA**C**CTG**G**AA*NGG | - | NC_010460.4 | 733598 |
| OTB4 (ABCC9) | **A**CTGCTTGTCTCAACT**A**T**TT** | - | NC_010447.5 | 100127449 |
| OTB5 (ACOXL) | **C**CTGCTTGTCTCAACT**C**T**G**A | - | NC_010445.4 | 100520554 |
| OTB6 (ASIP) | **C**CTGCTTG**G**CTC**T**ACT**CC**T**T** | + | AB206998.1 | 414439 |
| OTB7 (HIPK3) | **A**CTGCTTG**G**CTC**T**ACT**T**T**G**A | - | NC_010444.4 | 100515770 |
| OTB8 (HHIP) | **CA**TGCTTGT**GA**CAACTG**ATG** | + | NC_010450.4 | 100525059 |
