# Supplementary material for: Optimizing sgRNA length to improve target specificity and efficiency for the GGTA1 gene using the CRISPR/Cas9 gene editing system
Source: PLoS One. 2019 Dec 10;14(12):e0226107. doi: 10.1371/journal.pone.0226107 (PMC6903732; doi:10.1371/journal.pone.0226107)
Supplement: S2 Table — (DOCX) [file pone.0226107.s004.docx]

**S2 Table.**

| Genes | Primer sequence (5’-3’) | PCR Product Size (bp) | Tm (°C) | Reference/Sequence Accession No. |
| --- | --- | --- | --- | --- |
| GGTA1 F | CCTTAGCGCTCGTTGACTATTC | 586 | 56 | NC_010443.5 |
| GGTA1 R | TTTCTTTGCTTTTTAGGGCCGC | 586 | 56 | NC_010443.5 |
| OTB1-GDE1 F | GATGGGAACTACGGGTTCTTT | 455 | 58 | NC_010445.4 |
| OTB1-GDE1 R | TCAGGAAATGTGTCAGGAGAAG | 455 | 58 | NC_010445.4 |
| OTB2-TVP23A F | GGCTGTGTGAAGAGGACTAAAT | 857 | 58 | NC_010445.3 |
| OTB2-TVP23A R | GAGATGGACGATGGAAGACAAA | 857 | 58 | NC_010445.3 |
| OTB3-FSCN3 F | GGATGTGAGAAAGAGGGTCTAAG | 659 | 58 | NC_010460.4 |
| OTB3-FSCN3 R | GGAAGAGGGACACTCATTACAG | 659 | 58 | NC_010460.4 |
| OTB4-ABCC9 F | GAGAGAGACTGAGAAACGTTGG | 655 | 60 | NC_010447.5 |
| OTB4-ABCC9 R | CTACAAGCATGGGTGGTAGTT | 655 | 60 | NC_010447.5 |
| OTB5-ACOXL F | GGTGCTTGGACATAGGGTTAG | 497 | 60 | NC_010445.4 |
| OTB5-ACOXL R | CCACATCCCTGGCACTAATAA | 497 | 60 | NC_010445.4 |
| OTB6-ASIP agouti F | CTGACTCACCAAGTCCACTATC | 477 | 62 | AB206998.1 |
| OTB6-ASIP agouti R | GGAAACTCAAATGCCTCTTCTG | 477 | 62 | AB206998.1 |
| OTB7-HIPK3 F | CGGCCTAGAGGTTAAGGATTG | 639 | 60 | NC_010444.4 |
| OTB7-HIPK3 R | GTGTCCTCGAGGTCTGTTAAAT | 639 | 60 | NC_010444.4 |
| OTB8-HHIP F | CCTCGAGCTGAATTAGCAGAAA | 622 | 58 | NC_010450.4 |
| OTB8-HHIP R | GTGTAATCTGGGAGAGCTAGGA | 622 | 58 | NC_010450.4 |
